# Editorial

**Published:** 1981

**Authors:** 


### Editorial

### Sponsorship for Survival

Our last editorial on the state of the Journal ended with the words 'Transfusion will be needed'. A donor has been found and two units of well-matched cells have been received. *Smith, Kline & French Laboratories Ltd.* have come to our assistance and have donated funds sufficient to cover the cost of producing two numbers of the Journal. This transfusion is well matched because it is in part a recognition of basic research on the development of *Cimetidine* done in Bristol at Frenchay Hospital. It is also a gesture of encouragement and support from the Scientific Staff of a firm that includes three Bristol graduates among its top management.

Ultimately this journal must be self-financing on its advertising revenue, it has been so in the past and can become so again. This timely and generous gesture of support will start us back again on this road.

